# Does Usage of an eHealth Intervention Reduce the Risk of Excessive Gestational Weight Gain? Secondary Analysis From a Randomized Controlled Trial

**DOI:** 10.2196/jmir.6644

**Published:** 2017-01-09

**Authors:** Meredith Leigh Graham, Myla S Strawderman, Margaret Demment, Christine Marie Olson

**Affiliations:** ^1^ Division of Nutritional Sciences Cornell University Ithaca, NY United States; ^2^ Department of Obstetrics and Gynecology University of Rochester Rochester, NY United States

**Keywords:** Internet, obesity, weight gain, pregnancy

## Abstract

**Background:**

Excessive gestational weight gain (GWG) contributes to the development of obesity in mother and child. Internet-based interventions have the potential for delivering innovative and interactive options for prevention of excessive GWG to large numbers of people.

**Objective:**

The objective of this study was to create a novel measure of Internet-based intervention usage patterns and examine whether usage of an Internet-based intervention is associated with reduced risk of excessive GWG.

**Methods:**

The website featured blogs, local resources, articles, frequently asked questions (FAQs), and events that were available to women in both the intervention and control arm. Weekly reminders to use the website and to highlight new content were emailed to participants in both arms. Only intervention arm participants had access to the weight gain tracker and diet and physical activity goal-setting tools. A total of 1335 (898 intervention and 437 control) relatively diverse and healthy pregnant women were randomly assigned to the intervention arm or control arm. Usage patterns were examined for both intervention and control arm participants using latent class analysis. Regression analyses were used to estimate the association between usage patterns and three GWG outcomes: excessive total GWG, excessive GWG rate, and GWG.

**Results:**

Five usage patterns best characterized the usage of the intervention by intervention arm participants. Three usage patterns best characterized control arm participants’ usage. Control arm usage patterns were not associated with excessive GWG, whereas intervention arm usage patterns were associated with excessive GWG.

**Conclusions:**

The control and intervention arm usage pattern characterization is a unique methodological contribution to process evaluations for self-directed Internet-based interventions. In the intervention arm some usage patterns were associated with GWG outcomes.

**ClinicalTrial:**

ClinicalTrials.gov; Clinical Trials Number: NCT01331564; https://clinicaltrials.gov/ct2/show/NCT01331564 (Archived by WebCite at http://www.webcitation/6nI9LuX9w)

## Introduction

Maternal obesity and excessive gestational weight gain (GWG) are associated with many adverse pregnancy and birth outcomes, such as gestational diabetes and cesarean delivery, in addition to an increase in obesity risk in both mother and baby [1-3]. A recent Cochrane review found that diet and exercise interventions during pregnancy reduced the risk of excessive GWG by 20% [4]. This review suggested that electronic communications interventions may have potential for addressing these growing public health problems.

Electronic health (eHealth) interventions have the advantages of wide reach, interactivity, personalization, and cost-effectiveness. eHealth interventions have been shown to be efficacious across cognitive outcomes (knowledge, intention, and self-efficacy), some behavioral outcomes (smoking cessation, reducing alcohol consumption, safer sexual behaviors, and increasing physical activity), and emotional outcomes (mild to moderate depression, anxiety, obsessive-compulsive disorder, and phobias) [5]. A review and meta-analysis by Hill et al [6] found that providing information and behavioral self-monitoring were two key strategies when intervening in GWG. However, according to a recent review, there is a lack of clarity about the effectiveness of behavioral interventions to address maternal obesity and GWG and, in particular, there is a need to identify the specific intervention components that contribute to the effectiveness of these interventions [7].

Generally, higher dose of intervention received and greater use of intervention features have been associated with greater success in achieving weight-related intervention outcomes [8-10]. This has been particularly true for eHealth or Internet-based behavior change interventions [11-13]. While Internet-based interventions provide a unique opportunity to measure use of behavior change tools and other features objectively, currently there is no consensus on the definitions and measures for usage of such interventions [14]. Previous studies have used the following measures: number of website visits or log-ins, time spent on a site, and number of features used [14-16].

In a previously published article, we described the creation of measures of intervention use that considered expected use, consistency of use across time, and patterns of use for different features of an Internet-based intervention aimed at preventing excessive GWG [17]. This study examined whether the patterns of features that were used and the amount of their use were related to GWG outcomes among women participating in a randomized controlled trial. We used the previously described usage pattern measures for the intervention arm women, created a new usage measure for the control arm women, and then examined how these measures of usage were associated with 3 different GWG outcomes.

## Methods

### Study Design

Data from a randomized controlled trial of prevention of excessive GWG and postpartum weight retention with women who were 18-35 years of age, normal range to obese class I body mass index (BMI), socially and racially diverse, and relatively healthy (N=1689) and conducted in a midsize city in northeastern United States were used in this study. This trial, conducted from 2011 to 2014, is described in detail elsewhere [18,19], and its ClinicalTrials.gov identifier is NCT01331564. Pregnant women were screened in prenatal clinics, private obstetric practices, practices that provide ultrasounds, and over the phone and online. To meet eligibility criteria, participants had to (1) consent at or before 20 weeks’ gestation, (2) be available for a 24-month intervention, (3) plan to carry their pregnancy to term and keep their baby, (4) read and understand English, and (5) have an email address. Exclusion criteria included the following: BMI less than 18.5 kg/m^2^ (underweight) or greater than or equal to 35.0 kg/m^2^ (obese class II or greater), multiple gestation (eg, twins), having had eating disorders or gastric bypass surgery in the past, having had 3 or more consecutive miscarriages, and the presence of prepregnancy medical conditions that could influence weight loss or gain.

All study participants were sent an email describing the tools on the website. Email, postcard, and telephone reminders were used as prompts to encourage participants to visit the website the first time. A US $5 incentive was also given for the first website visit. The sample for this analysis includes women who were eligible, entered the study during pregnancy as indicated by at least one website log-in or completion of the baseline questionnaire, and had a singleton pregnancy that lasted at least 20 weeks (n=1335). The study protocol was approved by the University of Rochester Research Subjects Review Board and the Cornell University Institutional Review Board.

### Intervention

**Figure 1:**
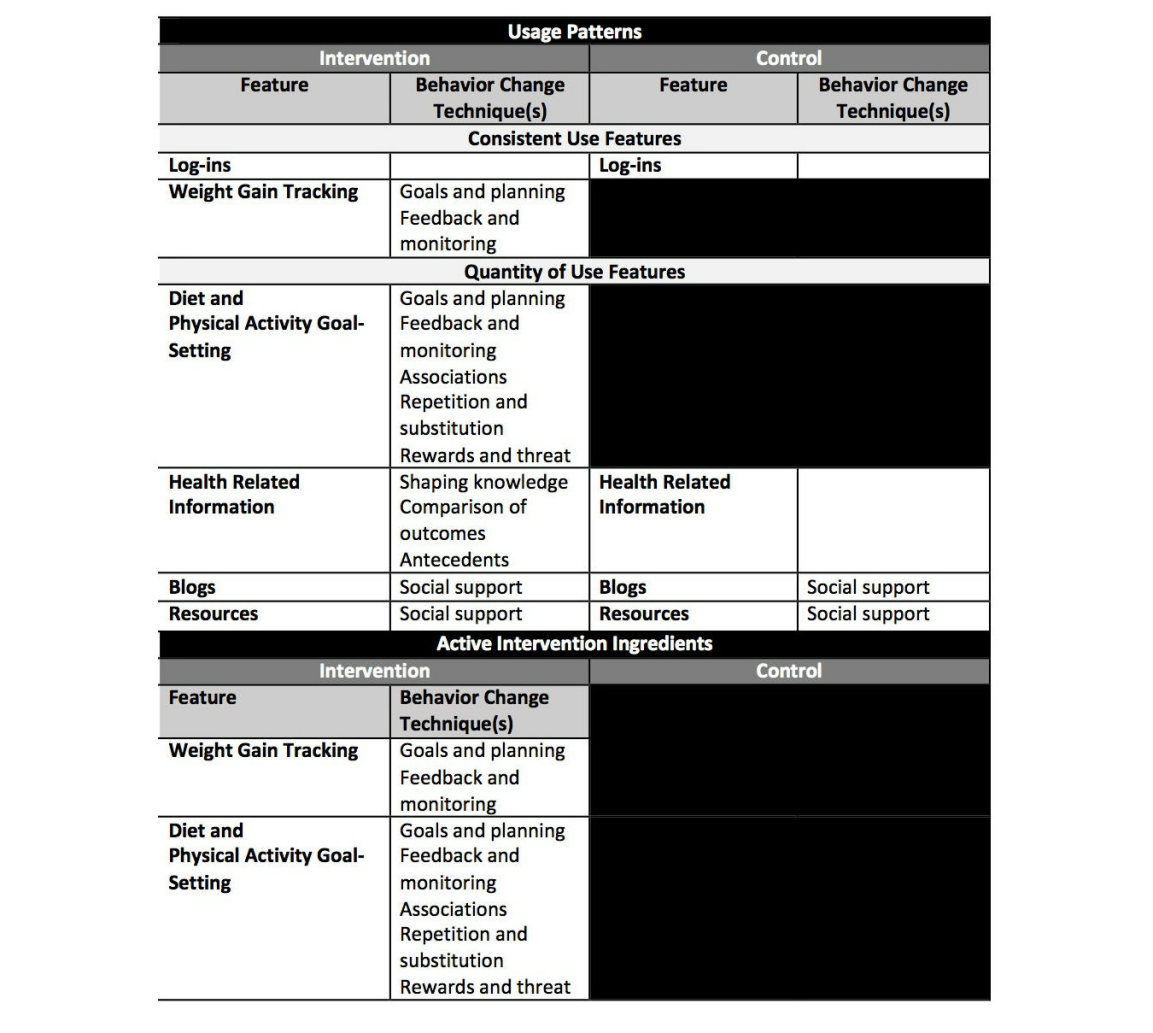
Website features, behavior change strategies, and expected use of intervention features.

Fishbein and Yzer’s Integrative Model of Behavioral Prediction [20] was the guiding theoretical framework for the Internet-based intervention to prevent excessive GWG. Fishbein and Yzer’s framework was combined with Fogg’s Behavior Model for Persuasive Design [21] to link weight-associated behaviors and their predictors to intervention features. Michie’s behavior change techniques [7,22] informed the development of the website features for the intervention arm (Figure 1). The website featured blogs, local resources, articles, frequently asked questions (FAQs), and events that were available to women in both the intervention and control arm. Weekly reminders to use the website and to highlight new content were emailed to participants in both arms. In addition, intervention arm participants had access to the weight gain tracker and diet and physical activity goal-setting tools. Intervention participants were emailed weekly with reminders to use the weight gain tracker and diet and physical activity goal-setting tools. Intervention features are described in more detail in the study by Graham et al [23].

### Consistent Use Features

**Figure 2:**
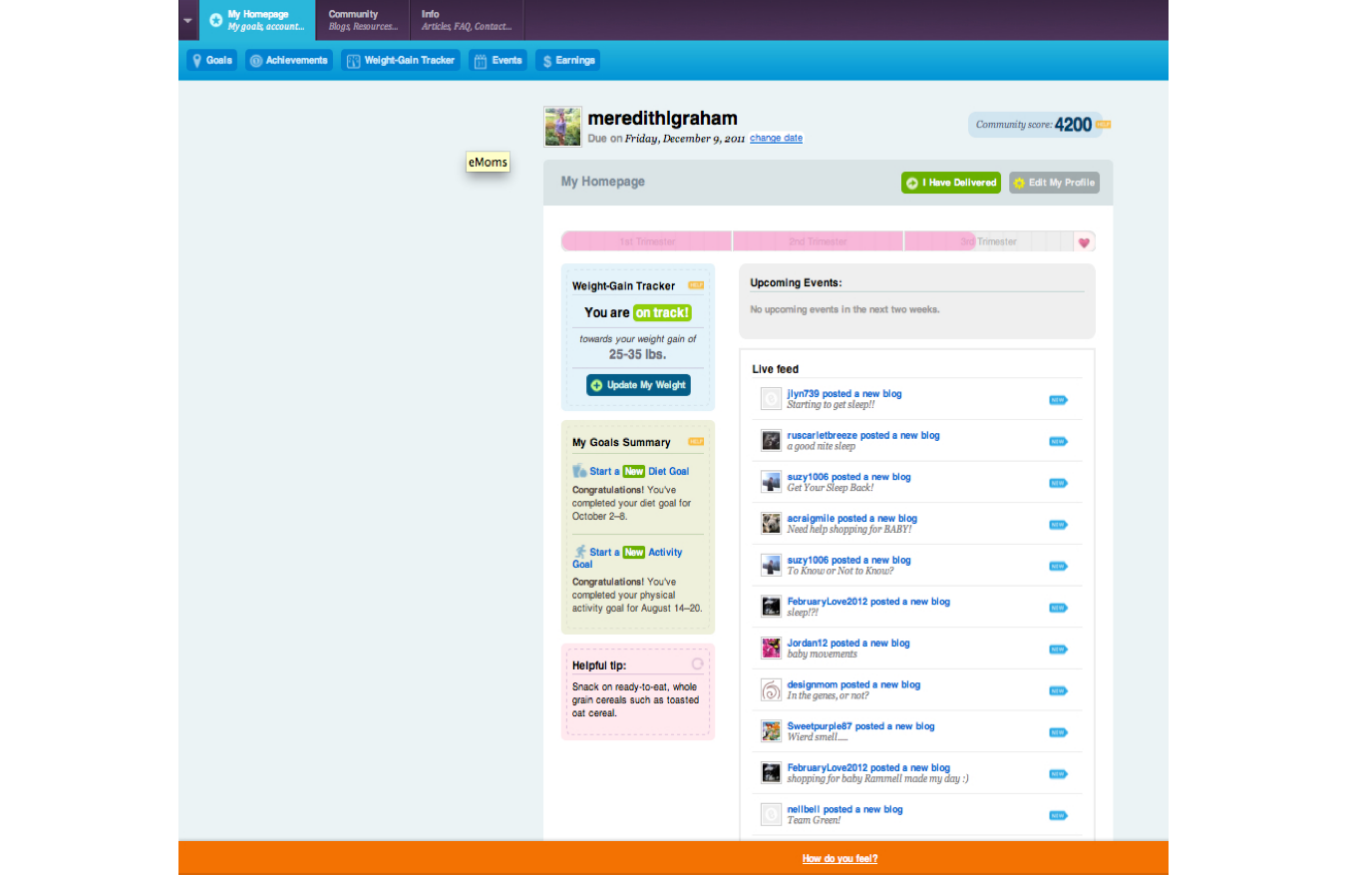
Screenshot of the intervention arm website dashboard.

**Figure 3:**
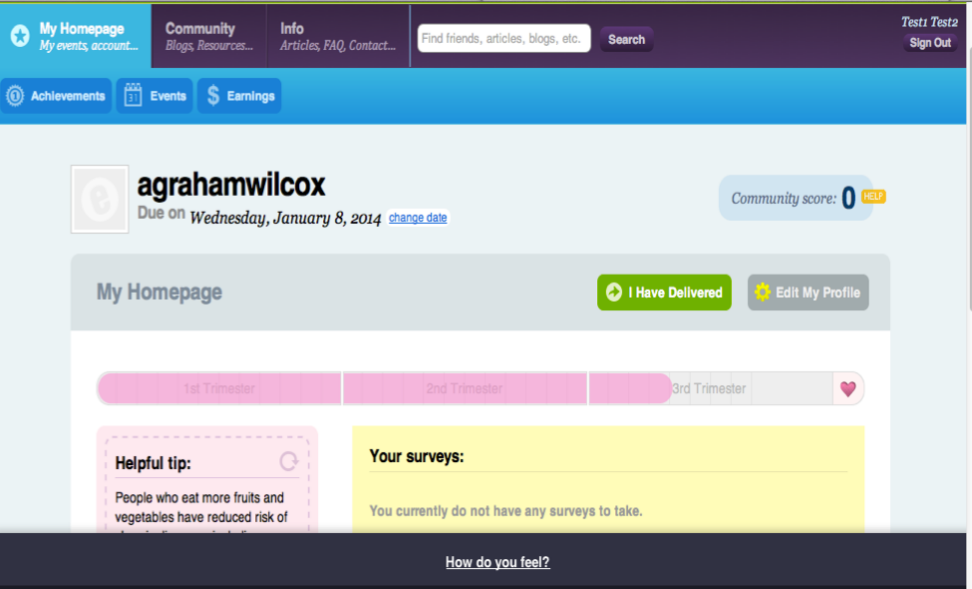
Screenshot of the control arm website dashboard.

Use of the Internet-based features was automatically captured by the website. Utilization of the following 6 intervention features plus log-ins was used to characterize usage in the intervention arm: health-related information (articles and FAQs), blogs, local resources, diet goal-setting tools, physical activity goal-setting tools, and a weight gain tracker (Figure 1). For the control arm, log-ins plus the use of the first 3 features mentioned above were included in the patterns-of-usage measure. For some features, the amount of use in relation to expected use was captured in the patterns-of-usage measure. Consistent use was expected for log-ins and entry of weights into the weight gain tracker. We expected women to track their weight in 30-day intervals, but, to allow for difference in timing of prenatal care, we created 45-day intervals from time of enrollment to delivery. If a woman entered a weight during each of the 45-day intervals of her study participation, she was categorized as a *consistent tracker*. If during at least half of the intervals a woman entered a weight, she was categorized as an *almost consistent tracker*. If a woman had entered at least one weight but not during more than half of her intervals, she was categorized as an *inconsistent tracker*. Finally, if she never entered a weight during pregnancy, she was categorized as a *nontracker*. We counted website log-ins as feature usage given the amount of content that was visible on the website dashboard after log-in for both intervention and control arm participants (Figures 2 and 3). The same procedure for consistent weight tracking was used to categorize log-ins.

### Quantity of Use Features

For all other features, consistent use was not expected. Use was expected on an “as needed” basis. Therefore, quantity of use was used for the following features: health-related information, blogs, resources, diet goal-setting tools, and physical activity goal-setting tools. The usage of a feature by a woman was categorized into 3 levels for each of these features: *high* (≥median among users), *low* (<median among users), or *none* (0).

### Usage Patterns

Latent class analysis (LCA) was used to identify usage patterns by women in the intervention arm and the control arm. This analysis was used to group individuals based on their similar usage patterns. All analyses were conducted using a SAS procedure, PROC LCA version 9.2 (SAS Institute). The creation of the intervention usage pattern variables is described in greater detail in the study by Demment et al [17]. The sample used in this analysis excluded women who had never logged in and had not completed a questionnaire, which influences the latent classes that emerge.

### Outcome Measure

GWG data were obtained through an audit of the participants’ prenatal, labor and delivery, and 6-week postpartum medical records. Total GWG was calculated as the difference between the first weight and the last weight in pregnancy. Overall, 12.80% (171/1335) of the sample did not have sufficient weight information in the prenatal chart to yield a valid measured GWG. Sufficient weight information required having a measured weight at both <14 weeks’ and ≥37 weeks’ gestation. Missing data were handled using multiple imputation to address issues of bias [24], which may result from analyzing only complete cases using SAS PROC MI and MIANALYZE procedures. A total of 60 imputed datasets were created for the primary analysis and also for the models presented below. The weekly rate of GWG was calculated as the difference between the last pregnancy weight and the weight measured closest to 20 weeks (±3 weeks), or the imputed average weight at 18-21 weeks if missing, divided by the number of weeks between these weights. Next, the binary outcomes of excessive total GWG and excessive rate of GWG after 20 weeks were calculated using the Institute of Medicine guidelines for each BMI group as determined at randomization. For each of the 3 BMI groups, the cutoff values for excessive total GWG and excessive weekly rate of GWG are as follows: normal range BMI, greater than 16 kilograms and greater than 0.23 kilograms per week; overweight BMI, greater than 11.5 kilograms and greater than 0.15 kilograms per week; and obese class I BMI, greater than 9 kilograms and greater than 0.12 pounds per week.

### Statistical Analysis

Among those participants with measured and available GWG data, chi-square analysis was used to examine the relationship between usage patterns and (1) demographics and (2) the binary outcomes of excessive total GWG and excessive rate of GWG. For total GWG, analysis of variance was used to examine the relationship between usage patterns and GWG. Since the created usage patterns differed by arm, these analyses were performed separately within intervention arm participants and within control arm participants.

Using a modified Poisson regression approach [25], the relative risk of excessive total or weekly GWG was estimated for different usage patterns within strata. Similarly, least squares multiple regression models assessed the mean difference in total GWG (kilograms). Usage was parameterized with two approaches. First, we examined by strata whether or not various patterns of usage were associated with relative risk of excessive GWG. Second, we examined by strata how the combined usage patterns that include most frequent usage of the active ingredients of the intervention, that is, weight gain tracking and behavioral goal setting and self-monitoring (Figure 1), were associated with relative risk of GWG. All models were adjusted for BMI, age, race, and parity, as well as pregnancy timing variables including gestational age at delivery, the weeks between the first and last pregnancy weight, and the weeks between the last pregnancy weight and delivery. Significance level was set at *P* ≤.05.

## Results

Usage pattern measures were created for each arm (Table 1). For the intervention arm, the 5-component solution was the best fit based on Akaike information criterion (AIC) score. All 5 usage patterns included consistent or almost consistent logging in. The 5 patterns and their components are as follows: (1) super user—consistent weight tracking; very likely to be a high user of blogs, health-related information, and local resources; likely to be a high user of diet or physical activity goal setting; (2) medium user—almost consistent weight tracking; likely to be a high user of blogs, health-related information, local resources, and physical activity goal setting; (3) consistent tracker—likely consistent weight tracking; likely low user of health-related information and blogs; unlikely to set goals or view resources; (4) almost consistent or inconsistent tracker—likely almost consistent weight tracker (although 38% likelihood of also being an inconsistent weight tracker); unlikely to have used goal-setting tools, health-related information, or local resources; unlikely to have used blogs; and (5) “nonuser”—almost consistent log-ins (likely adherent study participant); unlikely to have tracked weight; very unlikely to have used any other intervention feature.

For the control arm, the 3-group solution was the best fit based on the AIC score. The 3 patterns and their components are as follows: (1) mid or high user—very likely to consistently log in; very likely to be a high user of health-related information, blogs, and local resources; (2) low user—likely to have logged in consistently or almost consistently; likely to have been a high user of blogs (although 37% likelihood of also never viewing blogs), but unlikely to have viewed any local resources; and (3) no or minimal user—very likely to have logged in almost consistently or inconsistently; unlikely to have viewed health-related information, blogs, or local resources.

Within the intervention arm participants, there were significant demographic differences by patterns of usage (Table 2). Higher usage groups, including consistent trackers, were more likely to have higher income, be white, be older, and have ever been married. There are also differences in GWG outcomes by patterns of usage (Table 2). Nonusers and consistent trackers were least likely to exceed the recommended amount of GWG. Almost consistent or inconsistent trackers were most likely to exceed the recommended amount. Nonusers, consistent trackers, and super users have the lowest amount of total GWG.

**Table 1:** Usage patterns latent class probablities.

Usage pattern		Intervention (n=898)					Control (n=437)		
		Super user, 138 (15.4%)	Medium user, 89 (9.91%)	Consistent tracker, 181 (20.2%)	Almost consistent or inconsistent tracker, 275 (30.6%)	Nonuser, 215 (23.9%)	Mid or high user, 164 (37.5%)	Low user, 157 (35.9%)	No or minimal user, 116 (26.5%)
**Log-in**								
	Consistent	*.94*^a^	.005	*.97*	.06	.03	*.88*	*.42*	.007
	Almost consistent	.06	*.96*	.03	*.92*	*.79*	.12	*.58*	*.93*
	Inconsistent	.0001	.04	.0001	.02	.17	0	0	.06
	Never	0	0	0	0	0	0	0	0
**Weight tracking**								
	Consistent	*.86*	.002	*.71*	.007	.009			
	Almost consistent	.13	*.80*	.23	*.48*	.10			
	Inconsistent	.009	.13	.03	.38	.28			
	Never	.0004	.07	.03	.13	*.61*			
**PA^b^ goal setting**								
	High	*.47*	*.51*	.16	.06	.0002			
	Low	.27	.15	.16	.28	.03			
	None	.29	.34	*.68*	*.66*	*.97*			
**Diet goal setting**								
	High	*.47*	.38	.15	.11	.0002			
	Low	.18	.20	.20	.28	.03			
	None	.34	.42	*.65*	*.62*	*.97*			
**Health-related information**								
	High	*.81*	*.74*	.18	.11	.006	*.89*	.38	.02
	Low	.19	.22	*.46*	.40	.05	.06	.31	.06
	None	.001	.04	.36	*.49*	*.94*	.04	.31	*.93*
**Blogs**								
	High	*.93*	*.81*	.24	.17	.001	*.87*	*.44*	.03
	Low	.06	.15	*.43*	*.49*	.09	.09	.20	.09
	None	.02	.05	.34	.34	*.91*	.05	.37	*.89*
**Local resources**								
	High	*.87*	*.60*	.06	.05	.003	*.87*	.20	.005
	Low	.13	.27	.33	.25	.04	.11	.17	.04
	None	.003	.12	*.61*	*.70*	*.96*	.02	*.63*	*.95*

^a^Italics denotes probability greater than or equal to .40.

^b^PA: physical activity.

**Table 2:** Intervention arm: demographic and outcome differences by usage pattern (n=898).

Demographics		Super user, n (%)	Medium user, n (%)	Consistent tracker, n (%)	Almost consistent or inconsistent tracker, n (%)	Nonuser, n (%)
No. of participants		138 (15.4)	89 (9.9)	181 (20.2)	275 (30.6)	215 (23.9)
**Income (*P* <.001)**						
	Low income	29 (21.0)	32 (36.0)	52 (28.7)	118 (42.9)	95 (44.2)
	Not low income	109 (79.0)	57 (64.0)	129 (71.3)	157 (57.1)	120 (55.8)
**BMI^a^ at screening (*P*=.04)**						
	Normal range BMI	79 (57.3)	50 (56.2)	106 (58.6)	152 (55.3)	110 (51.2)
	Overweight BMI	34 (24.6)	30 (33.7)	56 (30.9)	90 (32.7)	59 (27.4)
	Obese BMI	25 (18.1)	9 (10.1)	19 (10.5)	33 (12.0)	46 (21.4)
**Strata (*P* <.001)**						
	Normal weight and lower income	10 (7.2)	17 (19.1)	30 (16.6)	62 (22.5)	60 (27.9)
	Normal weight and higher income	69 (50.0)	32 (36.0)	77 (42.5)	91 (33.1)	50 (23.3)
	Overweight or obese and lower income	19 (13.8)	15 (16.9)	22 (12.2)	56 (20.4)	60 (27.9)
	Overweight or obese and higher income	40 (29.0)	25 (28.1)	52 (28.7)	66 (24.0)	45 (20.9)
**Race (*P*<.001)**						
	Black	6 (4.3)	14 (15.7)	21 (11.6)	54 (19.6)	87 (40.5)
	White	118 (85.5)	64 (71.9)	138 (76.2)	180 (65.5)	92 (42.8)
	Other	14 (10.2)	11 (12.4)	22 (12.2)	41 (14.9)	36 (16.7)
**Hispanic (*P*=.08)**						
	Yes	13 (9.4)	5 (5.6)	20 (11.0)	33 (12.0)	35 (16.3)
	No	125 (90.6)	84 (94.4)	161 (89.0)	242 (88.0)	180 (83.7)
**Relation group (*P*<.001)**						
	Single	33 (23.9)	32 (36.0)	59 (32.8)	124 (45.4)	131 (61.8)
	Ever married	105 (76.1)	57 (64.0)	121 (67.2)	149 (54.6)	81 (38.2)
**Parity (*P*=.73)**						
	Nulliparous	71 (51.5)	46 (51.7)	79 (43.6)	127 (46.2)	93 (43.5)
	Primiparous	45 (32.6)	25 (28.1)	65 (35.9)	88 (32.0)	75 (35.0)
	Multiparous	22 (15.9)	18 (20.2)	37 (20.4)	60 (21.8)	46 (21.5)
**Age categories, years (*P* <.001)**						
	18 to 24	18 (13.0)	19 (21.3)	34 (18.8)	80 (29.1)	87 (40.5)
	25 to 30	41 (29.7)	31 (34.8)	67 (37.0)	93 (33.8)	67 (31.2)
	>30	79 (57.3)	39 (43.8)	80 (44.2)	102 (37.1)	61 (28.4)
**Outcomes**						
**Total GWG^b^ (*P*=.03, n=781)**						
	Exceeded recommended amount	59 (45.0)	39 (48.1)	64 (40.3)	132 (56.2)	70 (40.0)
	Did not exceed recommended amount	72 (55.0)	42 (51.9)	95 (59.7)	103 (43.8)	105 (60.0
**Rate of GWG (*P*=.09, n=795)**						
	Exceeded recommended rate	82 (62.6)	59 (71.1)	98 (59.4)	176 (73.0)	114 (65.1)
	Did not exceed recommended rate	49 (37.4)	24 (28.9)	67 (40.6)	65 (27.0)	61 (34.9)
GWG, kg (*P*=.03)^c^, mean (SD)		13.66 (4.59)	14.37 (5.22)	13.59 (5.33)	14.87 (5.85)	13.29 (5.88)

^a^BMI: body mass index.

^b^GWG: gestational weight gain.

^c^Analysis of variance test results shown; all other *P* values provided in the table are chi-square *P* values.

Within the control arm participants there were similar demographic differences by patterns of usage as observed in the intervention arm participants. The demographic differences between the usage patterns in the control arm were driven by the no or minimal user; when low and medium users were compared there were no significant differences by demographics (Table 3). In addition to the demographic differences, parity was also significant within the control arm, where nulliparous women were more likely to be higher users of the website. There were no significant differences in GWG outcomes by control arm patterns of usage.

Given that there were demographic differences by GWG outcomes (Multimedia Appendices 1 and 2) and that there were demographic and GWG differences by patterns of usage (Tables 2 and 3), adjusted models were needed to examine the independent effect of usage patterns on weight outcomes (Table 4). In each of these models, the comparison group was participants who had the nonuser usage pattern. In the intervention arm, among participants with lower income and normal range BMI (stratum 1), the relative risk of excessive GWG was 1.92 times higher for an almost consistent or inconsistent tracker compared with the nonuser. An inconsistent tracker gained 2.48 kg more than a nonuser. Among participants with normal range BMI and higher income (stratum 2), the consistent trackers had 0.67 relative risk of excessive weekly GWG rate compared with nonusers, after adjusting for differences in BMI, age, race, parity, and gestational age at delivery. Among overweight and obese higher-income participants (stratum 4), the consistent trackers gained 2.78 kg less than nonusers.

We also examined GWG outcomes and usage patterns in the control arm (Table 5). In each of these models, the comparison group is participants who had the nonuser usage pattern. There is only one significant result in the control arm when looking at usage pattern and GWG, which was among overweight and obese lower-income control participants (stratum 3) where the low user usage pattern had a relative risk of excessive weekly GWG rate 1.35 times that of nonusers.

In addition to examining patterns of usage created through LCA, we combined the latent classes that emerged that included high usage of the active ingredient intervention features, which were theorized to have the greatest likelihood of reducing the risk of excessive GWG: the super user and the consistent tracker groups. Specifically consistent weight tracking loaded at probability of >0.70 in both the super user and the consistent tracker group. In these models, the comparison group is participants who were in the medium user, almost consistent or inconsistent tracker, or the nonuser groups (Table 6). Among participants with normal range BMI and higher income (stratum 2), consistent trackers and super users had reduced relative risk of excessive GWG amount (relative risk =0.64) and weekly rate (relative risk =0.72). They also gained 1.49 kg less in total GWG. Among overweight and obese higher-income participants (stratum 4), consistent trackers and super users had reduced relative risk of excessive GWG amount (relative risk =0.87) and gained 2.17 kg less compared with those who used the active ingredients less.

**Table 3:** Control arm: demographic and outcome differences by usage pattern (n=437).

Demographics		Mid or high user, n (%)	Low user, n (%)	No or minimal user, n (%)
No. of participants		164 (36.7)	157 (37.1)	116 (26.2)
**Income (*P*<.001)**			
	Low income	54 (32.9)	52 (33.1)	62 (53.5)
	Not low income	110 (67.1)	105 (66.9)	54 (46.5)
**BMI^a^ at screening (*P*=.06)**			
	Normal range BMI	100 (61.0)	92 (58.6)	55 (47.4)
	Overweight BMI	51 (31.1)	42 (26.8)	46 (39.7)
	Obese BMI	13 (7.9)	23 (24.6)	15 (12.9)
**Strata (*P*=.002)**			
	Normal weight and lower income	27 (16.5)	28 (17.8)	31 (26.7)
	Normal weight and higher income	73 (44.5)	64 (40.8)	24 (20.7)
	Overweight or obese and lower income	27 (16.5)	24 (15.3)	31 (26.7)
	Overweight or obese and higher income	37 (22.5)	41 (26.1)	30 (25.9)
**Race (*P*<.001)**			
	Black	22 (13.4)	23 (14.6)	37 (31.9)
	White	120 (73.2)	116 (73.9)	60 (51.7)
	Other	22 (13.4)	18 (11.5)	19 (16.4)
**Hispanic (*P*=.55)**			
	Yes	19 (11.6)	14 (9.0)	15 (12.9)
	No	145 (88.4)	143 (91.0)	101 (87.1)
**Relation group (*P*<.001)**			
	Single	53 (32.3)	52 (33.3)	66 (57.9)
	Ever married	111 (67.7)	104 (66.7)	48 (42.1)
**Parity (*P*=.005)**			
	Nulliparous	88 (53.7)	86 (54.8)	44 (37.9)
	Primiparous	49 (29.9)	46 (29.3)	35 (30.2)
	Multiparous	27 (16.4)	25 (15.9)	37 (31.9)
**Age categories, years (*P*=.003)**			
	18 to <25	37 (22.6)	33 (21.0)	46 (39.6)
	25 to <30	55 (33.5)	57 (36.3)	38 (32.8)
	>30	72 (43.9)	67 (42.7)	32 (27.6)
**Outcomes**			
**Total gestational weight gain (*P*=.62)**			
	Exceeded recommended amount	62 (43.7)	72 (49.3)	43 (45.3)
	Did not exceed recommended amount	80 (56.3)	74 (50.7)	52 (54.7)
** Rate of gestational weight gain (*P*=.54)**			
	Exceeded recommended rate	103 (70.6)	104 (71.2)	63 (65.0)
	Did not exceed recommended rate	43 (29.4.1)	42 (28.8)	34 (36.0)
Gestational weight gain, kg (*P*=.17)^b^, mean (SD)		14.48 (5.07)	14.19 (5.05)	13.21 (5.60)

^a^BMI: body mass index.

^b^Analysis of variance test results shown; all other *P* values provided in the table are chi-square *P* values.

**Table 4:** Intervention arm: Are usage patterns associated with gestational weight gain?

Usage patterns^a^		Excessive total GWG^b^			Excessive GWG rate			GWG		
		RR^c^	95% CI	*P* value	RR	95% CI	*P* value	Estimate	95% CI	*P* value
**Stratum 1: normal range BMI^d^ and low income (N=179)**									
	Super user^e^	0.74	0.20 to 2.74	.65	0.53	0.21 to 1.34	.18	−0.69	−3.80 to 2.41	.66
	Medium user	1.51	0.67 to 3.41	.32	1.10	0.72 to 1.70	.65	2.27	−0.89 to 5.43	.16
	Consistent tracker	1.61	0.89 to 2.91	.11	1.24	0.86 to 1.77	.25	1.79	−0.45 to 4.02	.12
	Inconsistent tracker	1.92	1.18 to 3.14	.009	1.25	0.95 to 1.64	.12	2.48	0.63 to 4.33	.009
**Stratum 2: normal range BMI and higher income (n=319)**									
	Super user	0.91	0.50 to 1.68	.77	0.85	0.59 to 1.22	.38	−1.22	−2.82 to 0.39	.14
	Medium user	1.04	0.52 to 2.07	.91	1.12	0.77 to 1.61	.56	0.10	−1.67 to 1.88	.91
	Consistent tracker	0.63	0.34 to 1.16	.14	0.67	0.46 to 0.98	.04	−1.22	−2.91 to 0.46	.15
	Inconsistent tracker	1.33	0.79 to 2.25	.29	1.05	0.77 to 1.44	.75	0.47	−1.12 to 2.06	.57
**Stratum 3: overweight or obese BMI and low income (n=172)**									
	Super user	1.34	0.92 to 1.95	.13	1.25	0.98 to 1.60	.07	0.55	−2.84 to 3.93	.75
	Medium user	0.96	0.56 to 1.64	.87	0.95	0.62 to 1.45	.81	−0.58	−4.51 to 3.34	.77
	Consistent tracker	1.24	0.81 to 1.89	.32	1.11	0.80 to 1.54	.52	1.10	−2.30 to 4.49	.53
	Inconsistent tracker	1.02	0.70 to 1.50	.92	1.10	0.86 to 1.42	.45	0.44	−2.06 to 2.93	.73
**Stratum 4: overweight or obese BMI and high income (n=228)**									
	Super user	0.96	0.72 to 1.30	.80	1.02	0.84 to 1.24	.83	−1.03	−3.59 to 1.53	.43
	Medium user	1.13	0.85 to 1.51	.41	1.06	0.85 to 1.31	.60	−0.01	−2.84 to 2.82	.99
	Consistent tracker	0.96	0.72 to 1.27	.76	1.01	0.83 to 1.23	.93	−2.78	−5.16 to −0.39	.02
	Inconsistent tracker	1.17	0.92 to 1.48	.21	1.13	0.95 to 1.34	.16	0.35	−1.94 to 2.64	.77

^a^All models have been adjusted for age, race, parity, and BMI.

^b^GWG: gestational weight gain.

^c^RR: relative risk.

^d^BMI: body mass index.

^e^Relative risk of excessive GWG and mean GWG estimates (kg) are relative to subjects who were nonusers (reference group).

**Table 5:** Control arm: Are usage patterns associated with gestational weight gain?

Usage patterns^a^		Excessive total GWG^b^			Excessive GWG rate			GWG		
		RR^c^	95% CI	*P* value	RR	95% CI	*P* value	Estimate	95% CI	*P* value
**Stratum 1: normal range BMI^d^ and low income (n=86)**										
	Low user^e^	1.30	0.64 to 2.64	.47	1.07	0.69 to 1.67	.76	−0.05	−2.88 to 2.77	.97
	Mid or high user	1.56	0.72 to 3.38	.26	1.25	0.79 to 2.00	.34	2.56	−0.10 to 5.22	.06
**Stratum 2: normal range BMI and higher income (n=161)**										
	Low user	0.83	0.40 to 1.75	.63	0.97	0.63 to 1.50	.90	−0.29	−2.24 to 1.66	.77
	Mid or high user	0.94	0.47 to 1.87	.85	1.13	0.75 to 1.72	.55	0.64	−1.36 to 2.64	.53
**Stratum 3: overweight or obese BMI and low income (n=82)**										
	Low user	1.31	0.81 to 2.13	.28	1.35	1.00 to 1.82	.05	2.31	−0.79 to 5.40	.14
	Mid or high user	1.11	0.67 to 1.82	.69	1.03	0.72 to 1.46	.89	1.39	−1.78 to 4.56	.39
**Stratum 4: overweight or obese BMI and high income (n=108)**										
	Low user	1.34	0.90 to 2.00	.15	1.09	0.84 to 1.43	.52	0.54	−2.08 to 3.16	.69
	Mid or high user	1.13	0.75 to 1.70	.56	1.12	0.86 to 1.47	.40	−0.44	−3.19 to 2.30	.75

^a^All models have been adjusted for age, race, parity, and BMI.

^b^GWG: gestational weight gain.

^c^RR: relative risk.

^d^BMI: body mass index.

^e^Relative risk of excessive GWG and mean GWG estimates (kg) are relative to subjects who were nonusers (reference group).

**Table 6:** Intervention arm: Are grouped usage patterns (super user and consistent tracker groups combined) associated with gestational weight gain after adjustment for demographics?

Usage patterns^a^	Excessive total GWG^b^			Excessive GWG rate			GWG		
	RR^c^	95% CI	*P* value	RR	95% CI	*P* value	Estimate	95% CI	*P* value
Stratum 1: normal range BMI^d^ and lower income (n=179)^e^	0.92	0.59 to 1.44	.71	0.93	0.69 to 1.26	.65	−0.32	−2.08 to 1.43	.71
Stratum 2: normal range BMI and higher income (n=319)	0.64	0.45 to 0.90	.01	0.72	0.57 to 0.90	.004	−1.49	−2.44 to −0.54	.002
Stratum 3: overweight or obese BMI and lower income (n=172)	1.28	0.98 to 1.68	.07	1.14	0.94 to 1.38	.19	0.74	−1.56 to 3.05	.53
Stratum 4: overweight or obese BMI and higher income (n=228)	0.87	0.73 to 1.02	.09	0.94	0.84 to 1.06	.31	−2.17	−3.58 to −0.76	.003

^a^All models have been adjusted for age, race, parity, BMI, and timing variables.

^b^GWG: gestational weight gain.

^c^RR: relative risk.

^d^BMI: body mass index.

^e^Relative risk of excessive GWG and mean GWG estimates (kg) are relative to subjects who were either medium users, inconsistent trackers, or nonusers (reference group).

## Discussion

### Principal Findings

We applied a novel approach, LCA, to understand usage of website features included in a GWG eHealth intervention. We examined patterns of usage for both the intervention participants and the control arm participants. This approach is a unique methodological contribution to process evaluations for self-directed Internet-based interventions where the most appropriate measures of engagement are not yet well defined. Usage patterns for both intervention and control arm participants varied by demographic characteristics. Higher-income, older, white, and married women in both arms were more likely to be higher users of the website. In the control arm, where the content of the website was primarily informational, women who were having their first baby were greater users.

In the control arm, GWG outcomes did not differ by usage pattern. While this was expected because behavior change and weight management tools were not included on the control website, documenting that lack of effect by amount of use helps in interpreting the relationship between amount of use of features in the intervention arm and weight outcomes. The concern is that amount of use is associated with a personality type that will have better outcomes, no matter what the content of the intervention is. The control group results indicate that this concern is likely not relevant to this study.

In the intervention arm, GWG outcomes did differ by usage pattern. Among participants with lower income and normal range BMI (stratum 1), almost consistent or inconsistent trackers had a higher relative risk of excessive GWG and inconsistent trackers gained more weight during pregnancy compared with the nonuser usage pattern. Among participants with normal range BMI and higher income (stratum 2), the consistent trackers had a lower relative risk of excessive weekly GWG rate compared with nonusers. Among overweight and obese higher-income participants (stratum 4), the consistent trackers gained less weight during pregnancy than nonusers.

In order to better understand the patterns of usage and GWG outcomes, we examined 2 of the usage groups together that included most frequent usage of the active ingredients of the intervention, weight gain tracking and behavioral goal setting and self-monitoring. We compared super users and consistent trackers with the 3 usage groups that used less of the hypothesized active ingredients in the pregnancy intervention (medium users, almost consistent or inconsistent trackers, and nonusers). In the higher-income stratum (strata 2 and strata 4), higher users of the active intervention ingredients were associated with reduced risk of excessive GWG total and in the normal range BMI women (stratum 2) for weekly rate. Across BMI categories, total GWG (kilogram) was significantly lower in the super and consistent users compared with the medium users, almost consistent or inconsistent trackers, and nonusers.

### Strengths and Limitations

The strengths of this study are as follows: the intervention’s measures of usage are objectively measured by the website and as such there is no study staff reporting bias for the intervention use variables; a large randomized effectiveness trial with an economically and racially diverse sample; a theory-based and formative research-informed Internet-based intervention.

A limitation of this research is that consistent weight tracking and logging in to the website was low and was particularly low among lower-income participants, with only 25% of low-income participants in either the consistent tracker or super user usage patterns. This affects the ability to detect statistically significant differences in the GWG outcomes between groups defined by usage.

### Implications

This study used a novel, data-driven approach to process evaluations that may be particularly helpful for self-directed Internet-based interventions on any topic. This approach may further the understanding of how self-directed Internet-based intervention tools are used and whether there are benefits associated with different patterns of use. The implications for this particular self-directed Internet-based intervention to prevent excessive GWG vary by socioeconomic status of the women. For higher-income women there was a reduction in GWG, but not necessarily a significant reduction in rate or excessive GWG for overweight or obese higher-income women. For lower-income women there were no detectable effects of usage on GWG. Future self-directed Internet-based interventions should consider best approaches for consistently engaging lower-income women when the success of interventions is anticipated to depend on consistent use.

## Appendix

Multimedia Appendix 1 Demographic differences by excessive gestational weight gain (intervention arm).

Multimedia Appendix 2 Demographic differences by excessive gestational weight gain (control arm).

## Abbreviations

AIC: Akaike information criterion
BMI: body mass index
FAQ: frequently asked question
GWG: gestational weight gain
LCA: latent class analysis
