# Supplementary material for: Does Usage of an eHealth Intervention Reduce the Risk of Excessive Gestational Weight Gain? Secondary Analysis From a Randomized Controlled Trial
Source: J Med Internet Res. 2017 Jan 9;19(1):e6. doi: 10.2196/jmir.6644 (PMC5259588; doi:10.2196/jmir.6644)
Supplement: Multimedia Appendix 1 [file jmir_v19i1e6_app1.pdf]

Demographic Differences by Excessive Gestational Weight Gain (Intervention Arms) [a]

	**Excessive Total (N=781)**	**Excessive Rate (N=795)**	**Gestational weight gain (kg)** [c]
	364 (47%)	529 (67%)	Mean (SD)
**Income**	*P*=.79 [b]	*P*=.43	*P*=.01
Low Income	132 (46%)	202 (68%)	13.4 (6.4)
Not Low Income	232(47%)	327 (66%)	14.4 (4.9)
**BMI at screening**	*P*<.0001	*P*<.0001	*P*<.0001
Normal BMI	141 (32%)	250 (56%)	14.5 (4.8)
Overweight BMI	154 (66%)	193 (81%)	14.1 (5.7)
Obese BMI	69 (62%)	86 (78%)	11.5 (7.0)
**Strata**	*P*<.0001	*P*<.0001	*P*<.0001
Normal/Low Income	55 (39%)	97(64%)	14.9 (5.3)
Normal/Higher Income	86 (29%)	152 (51%)	14.4 (4.5)
Overweight or Obese/Low Income	77 (54%)	105 (73%)	11.8 (6.9)
Overweight or Obese/Higher Income	146 (74%)	175 (86%)	14.3 (5.5)
**Race**	*P*=.67	*P*=.82	*P*=.002
Other	47 (45%)	70 (65%)	13.5 (5.9)
Black	61 (44%)	101 (67%)	12.7 (6.3)
White	256 (48%)	358 (66%)	14.4 (5.2)
**Hispanic**	*P*=.11	*P*=.25	*P*=.0072
Yes	331 (48%)	55 (61%)	12.5 (6.1)
No	33 (38%)	474 (67%)	14.2 (5.4)
**Relation group**	*P*=.47	*P*=.15	*P*=.91
single	150 (49%)	227 (71%)	14.0 (6.3)
ever married	213 (45%)	300 (64%)	14.0 (5.0)
**Parity**	*P*=0.006	*P*=.0006	*P*<.0001
nulliparous	184 (52%)	264 (73%)	15.1 (5.3)
primaparous	121 (46%)	171 (64%)	13.6 (5.1)
multiparous	59 (37%)	94 (57%)	12.2 (6.2)
**Age Categories**	*P*=.24	*P*=.0003	*P*=.70
18 - <25	96 (51%)	152 (76%)	14.3 (6.3)
25 - <30	124 (48%)	171 (66%)	14.0 (5.6)
> 30	144 (43%)	206 (62%)	13.9 (4.9)

[a] Only measured data included in this table

[b] Chi-square p-values shown

[c] ANOVA test results shown
