# Supplementary material for: Does Usage of an eHealth Intervention Reduce the Risk of Excessive Gestational Weight Gain? Secondary Analysis From a Randomized Controlled Trial
Source: J Med Internet Res. 2017 Jan 9;19(1):e6. doi: 10.2196/jmir.6644 (PMC5259588; doi:10.2196/jmir.6644)
Supplement: Multimedia Appendix 2 [file jmir_v19i1e6_app2.pdf]

Demographic Differences by Excessive Gestational Weight Gain (Control Arm) [a]

	**Excessive total** (N=383)	**Excessive rate** (N=389)	**Gestational weight gain (kg)** [c]
	177 (46%)	270 (69%)	Mean (SD)
**Income**	*P*=.61[b]	*P*=.88	*P*=.30
Low Income	68 (48%)	100 (69%)	14.4 (6.0)
Not Low Income	109 (45%)	170 (70%)	13.8 (4.7)
**BMI at screening**	*P*<.0001	*P* <.0001	*P* =.003
Normal BMI	73 (34%)	135 (61%)	14.8 (4.7)
Overweight BMI	73 (61%)	94 (79%)	13.4 (5.8)
Obese BMI	31 (67%)	41 (87%)	12.3 (5.6)
**Strata**	*P* <.0001	*P* =.0001	*P* =.004
Normal/Low Income	28 (38%)	48 (62%)	15.6 (5.8)
Normal/Higher Income	45 (32%)	87 (60%)	14.4 (3.9)
Ovwt or Obese/Low Income	40 (59%)	52 (76%	13.1 (6.0)
Ovwt or Obese/Higher Income	64 (65%)	83 (85%)	13.1 (5.5)
**Race**	*P* =.11	*P* =.75	*P* =.79
Other	20 (39%)	34 (65%)	14.1 (5.5)
Black	35 (57%)	43 (68%)	14.5 (6.2)
White	122 (45%)	193 (70%)	14.0 (4.9)
**Hispanic**	*P* =.10	*P* =.87	*P* =.36
Yes	25 (58%)	31 (70%)	14.7 (5.6)
No	152 (45%)	239 (69%)	14.0 (5.2)
**Relation group**	*P* =.04	*P* =.25	*P* =.77
single	73 (51%)	108 (73%)	14.2 (5.9)
ever married	103 (42%)	162 (67%)	14.0 (4.8)
**Parity**	*P* =.36	*P* =.11	*P* =.003
nulliparous	93 (48%)	146 (74%)	14.9 (5.3)
primaparous	45 (41%)	71 (63%)	12.9 (4.5)
multiparous	39 (49%)	53 (66%)	13.7 (5.5)
**Age Categories**	*P* =.25	*P* =.09	*P* =.09
18 - <25	49 (51%)	71 (72%)	14.9 (5.7)
25 - <30	65 (49%)	101 (75%)	14.2 (5.3)

	**Excessive total** (N=383)	**Excessive rate** (N=389)	**Gestational weight gain (kg)** [c]
> 30	63 (41%)	98 (63%)	13.4 (4.8)

[a] Only measured data included in this table

[b] Chi-square p-values shown

[c] ANOVA test results shown
